# Childhood outcomes of fetal genomic copy-number variants: The prenatal microarray cohort study

**DOI:** 10.1016/j.gimo.2025.103464

**Published:** 2025-10-13

**Authors:** Jacqui McCoy, Cecilia Pynaker, Sharon Lewis, David J. Amor, Fiona Norris, Lucy Gugasyan, George McGillivray, Susan Fawcett, Matthew Regan, Joanne M. Said, Lisa Begg, Natasha Frawley, Nicola Yuen, Ron Wapner, Brynn Levy, Susan P. Walker, Jane Halliday, Lisa Hui

**Affiliations:** 1 Reproductive Epidemiology group, Murdoch Children’s Research Institute, Parkville, VIC, Australia; 2 Department of Paediatrics, University of Melbourne, Parkville, VIC, Australia; 3 Mercy Perinatal; Mercy Hospital for Women, Heidelberg, VIC, Australia; 4 Neurodisability and Rehabilitation group, Murdoch Children’s Research Institute, Parkville, VIC, Australia; 5 Victorian Clinical Genetics Services, Royal Children’s Hospital, Parkville, VIC, Australia; 6 Monash Health Pathology, Monash Medicine Centre, Clayton, VIC, Australia; 7 The Royal Women’s Hospital, Parkville, VIC, Australia; 8 Monash Genetics, Monash Health, Monash Medicine Centre, Clayton, VIC, Australia; 9 Department of Obstetrics, Gynaecology and Newborn Health, University of Melbourne, Parkville, VIC, Australia; 10 Department of Maternal Fetal Medicine, Joan Kirner Women’s and Children’s Sunshine Hospital, Western Health, St Albans, VIC, Australia; 11 Eastern Health, Box Hill Hospital, Box Hill, VIC, Australia; 12 Grampians Health, Ballarat Base Hospital, Ballarat, VIC, Australia; 13 Bendigo Health, Bendigo, VIC, Australia; 14 Columbia University Medical Center; 15 Northern Health, Epping, VIC, Australia

**Keywords:** Chromosomal microarray analysis, Cognitive function, Copy-number variants, Prenatal diagnosis, Variant of uncertain significance

## Abstract

**Purpose:**

The long-term developmental outcomes of children with a prenatal diagnosis of a copy-number variant of uncertain significance (VUS) remain unclear. This study compared the developmental, social-emotional, and health outcomes of children with and without a prenatal VUS, assessed maternal perceptions of their child’s health and development, and examined the reclassification rate of VUS after more than 2 years.

**Methods:**

Women who underwent prenatal chromosomal microarray testing in Victoria, Australia (2013-2019), were recruited retrospectively (2021-2023). Children with a VUS (cases) were compared with controls without a VUS. We assessed a range of cognitive, developmental, and health outcomes in the children, who were on average 6 years old. Statistical analyses compared group outcomes and adjusted for maternal sociodemographic factors.

**Results:**

The study included 134 mother-child pairs (46 with a VUS and 88 controls). No significant differences were found between groups in intellectual functioning, adaptive behavior, or social-emotional measures. Maternal perceptions of their child and family well-being were also similar. Reanalysis reclassified 66.0% of VUS as benign and 8.5% as pathogenic.

**Conclusion:**

Children with a prenatal VUS diagnosis have developmental outcomes and family well-being comparable to those without. These findings contribute valuable evidence to support prenatal genetic counseling and clinical laboratory reporting practices.

## Introduction

Over the past decade, advances in genomics have revolutionized reproductive health and prenatal diagnosis.^1^ Chromosomal microarray (CMA) has been the gold standard first line investigation for fetal structural anomalies since 2012^2^ and is commonly performed for all indications for prenatal diagnosis.^3^^,^^4^ Although CMA have enhanced the detection of pathogenic copy-number variants (CNVs), they also identify variants of uncertain significance (VUS).^5^ VUS can pose challenges for genetic counselling and decision-making in the prenatal setting^6^^,^^7^ because they may be associated with an increased chance of developmental disabilities or neuropsychiatric conditions.^8^ Unlike the postnatal context, in which uncertain genomic findings can be correlated with the child’s phenotype, the disclosure of fetal VUS during pregnancy can introduce significant uncertainty, increasing anxiety and raising ethical dilemmas regarding pregnancy management.^9^, ^10^, ^11^ Prenatal genetic counselling for VUS is further complicated by the systemic bias in the literature. Data on CNVs are mostly derived from symptomatic children, skewing the evidence base toward more severe phenotypes that may not be applicable in the prenatal context.^8^^,^^12^, ^13^, ^14^ Only 2 studies have investigated the outcomes of children born after a prenatal diagnosis of a CNV and neither examine health and development of children diagnosed with a VUS beyond 4 years.^15^^,^^16^

The paucity of comprehensive and long-term outcome data for children with a prenatally diagnosed VUS significantly impedes genetic counseling of expectant couples. We aimed to recruit a cohort of children, with and without a prenatal VUS, to (1) compare the developmental, social-emotional, and health status of children with and without a prenatal diagnosis of a VUS; (2) measure the impact of the CMA results on maternal perceptions of child health, behavior, and development; and (3) determine the proportion of prenatal VUS reclassified to benign or pathogenic on reanalysis > 2 years later.

## Materials and Methods

### Study design

We established a population-based cohort of mother-child pairs who had undergone prenatal testing with a CMA in Victoria, Australia between 2013 to 2019 inclusive. The full study protocol has been published elsewhere.^15^ Ethical and governance approval was granted by the Royal Children’s Hospital Human Research Ethics Committee (RCH HREC #60542) and the Mercy Health Human Research Ethics Committee (HREC #2020-046). The study was funded by the National Health and Medical Research Council (#1186862).

### Recruitment

Potential participants were identified from the Victorian Prenatal Diagnosis Database, a statewide research database of all prenatal chromosome testing results.^16^ During the study period, chromosome microarrays were used in >85% of prenatal diagnostic tests, including fetuses with and without structural anomalies.^17^ We screened potential cases from singleton pregnancies with a CNV reported on a prenatal microarray result. We defined cases as those with a VUS, inclusive of all indications for prenatal diagnosis including fetal ultrasound abnormality. Controls were singleton pregnancies that had no CNV (ie, no clinically significant genomic imbalance detected) detected on a prenatal microarray and no fetal ultrasound abnormality.

Medical records of potential cases and controls were screened for full eligibility criteria. Individuals were eligible if they (1) had a prenatal CMA result reported for a singleton pregnancy between January 2013 and December 2019, (2) were ≥18 years old at enrolment, (3) could provide informed consent in English, (4) had a live birth outcome, (5) were the primary caregiver at hospital discharge, (6) were able to consent on behalf of themselves and their child, (7) were able to complete questionnaires and attend study assessments, and (8) lived in Victoria. Participant recruitment and data collection occurred between November 2021 and December 2023. Full details of the recruitment process, perinatal outcomes, and participant response rate has been published elsewhere.^18^ In brief, 1832 medical records were screened and 1364 (75%) mother-child pairs met inclusion criteria. Of the 468 that did not meet inclusion criteria, 282 (60%) had “no live pregnancy outcome” (209 terminations of pregnancy (TOP), 73 miscarriages, stillbirths, and infant deaths), 157 (34%) required a translator, and 29 (6%) had other exclusion criteria. Approximately 77% of study invitation letters were successfully delivered by registered mail (1047/1364). The final recruitment rate was 19% (201/1047). All birth parents participating in this study identified as women/mothers; hence, these terms are used throughout.

### Outcome measures

#### Questionnaires

Demographic and health data were gathered at baseline using validated questionnaires. Mother-focused measures included the Patient Health Questionnaire,^19^ the State-Trait Anxiety Inventory,^20^ the Parenting Sense of Competence Scale,^21^ the McMaster Family Functioning Subscale,^22^ the Revised Scale for Ambiguity Tolerance,^23^ the Decision Satisfaction Scale,^24^^,^^25^ the Health Literacy Screening Questions,^26^ the Disclosure of Results to Others,^27^ the University of North Carolina Genomic Knowledge Scale,^28^ and 2 questions from the Genetics Essentialism scale.^29^^,^^30^ Child-focused measures included the Strengths and Difficulties Questionnaire,^31^ the Vulnerable Child Scale,^32^ and the Children with Special Health Care Needs Screener.^33^ A description of each questionnaire is available in Supplemental Table 1. Children aged 2 years and 6 months (2:6) or older were invited to participate in additional clinical assessments including validated cognitive, behavioral, social, and developmental assessments. These were conducted in person by psychologists and pediatricians.

#### Cognitive functioning

Age-appropriate Wechsler scales were used to assess intellectual functioning: the Wechsler Preschool and Primary Scale of Intelligence (WPPSI-IV) for children under 7 years and 7 months and the Wechsler Intelligence Scale for Children (WISC-V) for older children. Scores were classified according to test manual norms.^34^^,^^35^ Cognitive assessments for metropolitan participants were conducted in person at The Royal Children’s Hospital, whereas regional participants were assessed at local health services. Pediatricians and psychologists conducting assessments were initially blinded to the children’s CMA results. However, some parents spontaneously disclosed their experiences during assessments, despite being advised that assessing staff should remain blinded. Standardized assessments were consistently scored and either verified by a second team member or double-scored by the assessor.

#### Behavior and social functioning

The Vineland Adaptive Behavior Scales, Third Edition, Parent/Caregiver form (Vineland-3) assessed adaptive functioning across 5 domains: Communication, Socialization, Motor Skills, Daily Living Skills, and Maladaptive Behaviors (for children older than 3). Higher scores indicate better adaptive functioning.^36^

The Social Responsiveness Scale, Second Edition, Preschool/School Age forms (SRS-2) assessed social behaviors linked to autism spectrum disorder (ASD). Standardized scores categorized behaviors from normal to severe in relation to ASD symptoms.^37^

#### Child development

A pediatric review with the mother and child collected data on the child’s developmental and medical history, including growth, cognitive, behavioral, and neurological functioning, as well as any medical concerns using a standardized data collection form. Online pediatric assessments were available during COVID-19 pandemic restrictions in 2021 and 2022.

#### CNV reanalysis

The prenatal VUS classifications were reanalyzed by the original reporting laboratory. The scientists were blinded to the original classification and outcomes of the research assessments but were provided the indication for prenatal diagnostic testing. Reclassification was performed to assess the impact of advances in genomic knowledge, including updated databases, evolving guidelines, and emerging literature, on reporting practices. Reanalysis followed current best practice. Refer to Supplemental Methods for more detail.

### Statistical methods

Data were analyzed using Stata Version 18.^38^ Descriptive statistics included counts, percentages, means, standard deviations, and medians with interquartile ranges for nonnormally distributed data. Continuous outcomes were analyzed using *t* tests and multivariable linear regression for normally distributed data, whereas the Mann-Whitney test was used for nonnormally distributed data. Categorical outcomes were analyzed using the χ^2^ test. A significance threshold of *P* < .01 accounted for multiple comparisons. Scores from the 2 age-specific versions of the Strengths and Difficulties Questionnaire (for ages 2:0-3:11 and 4:0-10:0 years) were converted to *z*-scores and merged for further analysis. Sample sizes vary by measure because of differing participant completion rates across outcome measures.

## Results

**Figure 1:**
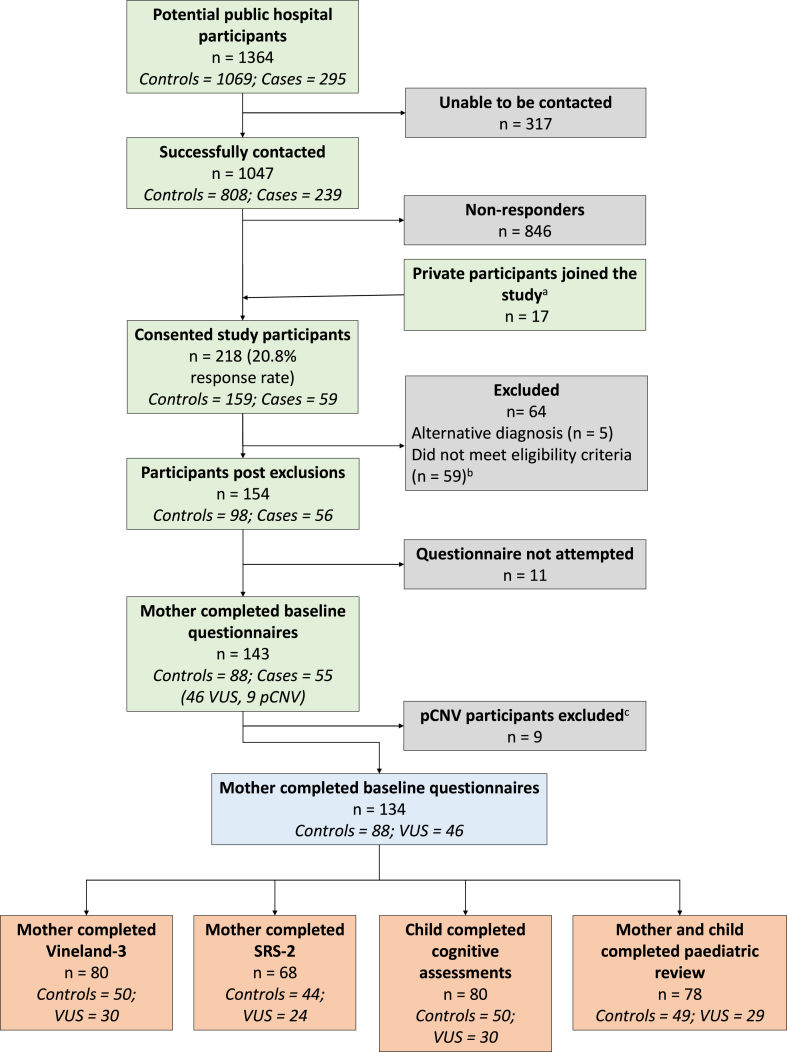
**Participant flow diagram.** A. An additional 17 participants were recruited from private clinical referrers, although the total number of screened private patients is unknown because of clinician-discretionary prescreening. B. Did not meet eligibility criteria after detailed review of medical record. C. Questionnaire and developmental assessment outcomes from 9 mother-child pairs with prenatally diagnosed pathogenic CNVs (pCNVs) were excluded in analyses because of small numbers and concerns regarding identifiability. CNV, copy-number variant; VUS, variant of uncertain significance.

From 1047 eligible mothers that were contacted, 218 (21%) consented to participate in the study (Figure 1). Fifty-nine participants were excluded after recruitment because of additional information obtained from the medical record, usually a finding of an ultrasound anomaly in the control group. The final analysis included 134 mother-child pairs, comprising 46 cases with a VUS and 88 controls (Figure 1).

#### Participant characteristics

A higher proportion of mothers in the control group were aged over 35 years at the time of giving birth compared with mothers in the VUS group (76%, versus 48%, respectively, *P* = .02). The VUS group also had a significantly higher proportion of regionally based participants compared with the control group (39% versus 19%, *P* = .01) due to study recruitment logistics (no controls were recruited from regional areas because of resource allocation). There were no other demographic differences between the VUS case and control cohorts, including those who completed only the online assessments (survey cohort) and those who opted for additional child assessments (clinical cohort, see Table 1).

**Table 1:** Demographic characteristics

Characteristic	Survey Cohort *n* = 134^a^					Clinical Cohort *n* = 78^a^				
	Controls (*n* = 88)		VUS (*n* = 46)		*P*	Controls (*n* = 49)		VUS (*n* = 29)		*P*
	*n*	% (col)	*n*	% (col)		*n*	% (col)	*n*	% (col)	
Maternal age at birth										
≥ 35 years	64	72.7	19	41.3	<.001	37	75.5	14	48.3	.02
Times pregnant										
≥ 3	50	57.5	23	50.0	.41	24	49.0	14	48.3	.95
Total number of children										
≥ 3	30	34.5	11	23.9	.21	13	26.5	7	24.1	.82
Number of people in household										
> 4	28	32.6	14	30.4	.80	12	24.5	7	24.1	.97
Relationship status										
Partnered	79	92.9	38	84.4	.12	45	93.8	23	82.1	.11
Not partnered	6	7.1	7	15.6		3	6.3	5	17.9	
Other/prefer not to answer	1		1			1		1		
Maternal highest education qualification										
Secondary	4	4.7	7	15.2	.11	2	4.1	4	13.8	.18
Diploma/certificate	23	26.7	12	26.1		10	20.4	8	27.6	
Tertiary	59	68.6	27	58.7		37	75.5	17	58.6	
Region										
Metro	71	80.7	28	60.9	.01	39	79.6	18	62.1	.09
Regional	17	19.3	18	39.1		10	20.4	10	37.9	
Paternal highest education qualification^b^										
Secondary	15	17.4	11	23.9	.48	10	20.4	6	20.7	.56
Diploma/certificate	27	31.4	12	26.1		12	24.5	9	31.0	
Tertiary	42	48.8	20	43.5		25	51.0	11	37.9	
Otherˆ	2	2.3	3	6.5		2	4.1	3	10.3	
Annual household income										
Up to $100,000	16	18.6	12	26.1	.42	9	18.4	8	27.6	.42
$100,001-150,000	29	33.7	13	28.3		15	30.6	8	27.6	
$150,001+	13	28.3	15	32.6		22	44.9	9	31.0	
Not sure/prefer not to say	6	7.0	6	13.0		3	6.1	4	13.8	
Born in Australia										
Yes	56	65.1	31	67.4	.79	30	61.2	22	75.9	.19
No	30	34.9	15	32.6		19	38.8	7	24.1	
Main language spoken at home										
English	70	81.4	40	87.0		40	81.6	26	89.7	
English and language other than English	6	7.0	1	2.2	.49	3	6.1	1	3.5	.64
Language other than English	10	11.6	5	10.9		6	12.2	2	6.9	

*VUS*, variant of uncertain significance.

a The survey cohort (*n* = 134) included mothers who completed baseline questionnaires, whereas the clinical cohort comprised mother-child pairs who completed pediatric reviews and additional assessments. Comparisons found no differences between those who completed only online assessments and those who underwent further assessments. Missing values ranged from 1 to 2.

b For some participants, the highest level of paternal education is unknown: the child was donor-conceived (*n* = 1), the respondent was unsure (*n* = 3), and others preferred not to answer (*n* = 2).

The most common indication for prenatal diagnosis among the pregnancies with a VUS was an ultrasound abnormality (57%, 26/46) (see Supplemental Tables 2 and 3 for details of the ultrasound abnormalities and perinatal outcomes). The most common indication for prenatal diagnosis among the controls was an increased probability of aneuploidy on the first trimester combined screen (48%, 42/88). Overall, 74% of the VUS cases were either maternally or paternally inherited.

Perinatal outcomes were compared between the VUS and control groups: there were no significant differences in sex ratio, head circumference, small for gestational age, mode of birth, or breast-feeding rates. The VUS group had significantly higher rates of special care nursery or neonatal intensive care admission and higher rates of hospital readmission within 28 days compared with controls (Supplemental Table 2).

### Developmental, social-emotional, and health outcomes

#### Cognitive functioning

General intellectual functioning was formally assessed in 80 out of 134 (60%) children with and without a VUS. The children’s average age at assessment was approximately 5 years and 11 months (range 2 years and 7 months to 9 years and 2 months). There was no significant difference in Full-Scale Intelligence Quotients (FSIQ) between children with a VUS (*n* = 30, mean 96.3, SD = 14.1) and those without (*n* = 50, mean 101.5, SD = 16.0; *P* = .14, see Table 2). Only 2 participants scored 2 SDs below the mean on the FSIQ (≤70), indicating mild intellectual impairment (1 each of VUS case and control).

**Table 2:** Measures of intellectual functioning, adaptive functioning, and behaviors associated with autism spectrum disorder

Measure (Population Mean [SD])	Domain (Higher Scores Indicate Higher Levels of the Domain Measured)	VUS (*n* = 30)^a^ Median (IQR)/Mean(SD)	Control (*n* = 50)^b^ Median (IQR)/Mean(SD)	*P*
WPPSI-IV / WISC-V (100 [15])	Overall intellectual functioning	96.3 (14.1)	101.5 (16.0)	.14
	Working memory	98.4 (16.6)	101.2 (15.8)	.47
	Visual-spatial reasoning	99.8 (14.6)	101.2 (14.9)	.70
	Verbal comprehension	95.5 (13.8)	103.7 (14.6)	.01
	Fluid reasoning	96.6 (17.7)	97.9 (15.6)	.75
	Processing speed	97.2 (13.5)	99.8 (14.8)	.49
Vineland-3 Parent/Caregiver form (100 [15])	Overall adaptive functioning	93.1 (13.9)	92.2 (10.5)	.73
	Communication	94.5 (82,102)	94.5 (88,100)	.93
	Daily Living Skills	93.7 (13.7)	92.9 (13.3)	.78
	Socialization	96.1 (13.9)	95.9 (10.1)	.94
	Motor skills (gross and fine)	96 (89, 102)	96 (89,104)	.88
Vineland-3 Parent/Caregiver form Maladaptive scales^c^	Internalizing behaviors (e.g., anxiety)	17 (15,18)	15 (14,18)	.61
	External behaviors (e.g., outbursts)	17 (14,19)	17 (14,18)	.41
SRS-2 (T-scores)^d^	Overall score of behaviors associated with Autism Spectrum Disorder	51.5 (46,59.5)	49 (43.5,55.5)	.20
	DSM-5 Compatible Scale: Social Communication and Interaction	52 (44.5,61)	48.5 (43.5,55.5)	.36
	DSM-5 Compatible Scale: Restricted Interests and Repetitive Behavior	48 (45.5, 59.5)	47 (43,55)	.25

*VUS*, variant of uncertain significance.

a The VUS sample size for each outcome ranged between *n* = 24-30.

b The Control sample size for each outcome ranged between *n* = 39-50.

c Maladaptive behaviors (Vineland-3)∗ v-Scale score 0-24 range; Mean = 15; SD = 3.

d ≤59 = within normal limits (generally not associated with autism spectrum disorder); between 60-65 = mild range; between 66-75 = moderate range; and ≥76 = severe range (strongly associated with clinical diagnosis of autism spectrum disorder).

A significant difference was observed on the Verbal Comprehension Index, in which children with a VUS (VUS cases; mean = 95.5, SD = 13.8) scored lower than children without (controls; mean = 103.7, SD=14.6; *P* = .01). However, this difference became nonsignificant when accounting for maternal age at the child’s birth, maternal country of birth, maternal education level, income, locality (metro/regional), and maternal relationship status (*P* = .08). No statistically significant differences were detected in the other cognitive indices after considering the same covariates (see Table 2, Supplemental Table 4).

#### Adaptive and social functioning

Eighty mothers (80/134; 60%) also completed the Vineland-3 Parent/caregiver form. There were no differences in overall adaptive functioning between VUS cases (mean = 93.1, SD = 13.9) and controls (mean = 92.2, SD = 10.5; *P* = .73). There were no differences observed between cases and controls in the other functional domains of the Vineland-3 (see Table 2). Sixty-eight (51%) mothers completed the SRS-2 questionnaire. There were no differences between VUS cases and controls observed in any of the social functioning scores (see Table 2).

#### Child development

A total of 78 (58%) mother-child pairs participated in the clinical review conducted by a study pediatrician. Of these, 26% (7/29 VUS cases and 13/49 controls) were seen in person, whereas the remainder were reviewed via Telehealth with video or telephone. No significant differences were observed between VUS cases and controls in the number of concerns reported across developmental and medical domains. The 2 exceptions were in the domains of infection and hearing, with more concerns reported in VUS cases than controls. For infection, specific issues within the VUS cohort included 2 cases of pyelonephritis, 7 cases of recurrent otitis media (≥3 episodes), and 3 classified as “other” recurrent types of infections. For hearing, there were 6 instances of conductive hearing loss and 1 case of sensorineural hearing loss reported among the VUS cases. See Supplemental Table 5 for detailed results of the pediatric review.

Body mass index was available for 70 (53%) children (42 controls and 28 VUS cases). There was no evidence of a difference in body mass index between the 2 groups, with 75% (21/28) of VUS cases and 60% (25/42, *P* = .34) of controls having a weight between the 5th and 85th percentiles standardized for age and sex.^39^

### Maternal perceptions of child health, behavior, and development

No group differences between maternal responses were found on measures of maternal mental health, perceived parental competence, overall family functioning, maternal perceptions of child behavior and vulnerability, general tolerance of uncertainty, general genetic knowledge, and decisional satisfaction regarding the choice to undergo microarray testing during pregnancy (see Supplemental Table 6).

There were no significant differences between the VUS and control groups in the proportion of children who were noted to receive at least 1 form of additional support, including medication, increased health or educational services, functional limitations, specialized therapies, or additional assistance for emotional, behavioral, or developmental issues (27% VUS vs 20% controls, *P* = .60). Health literacy was also similar, with 74% of VUS mothers and 77% of control mothers reporting no difficulty understanding written medical information, and 91% (VUS cases = 42; controls = 79) in both groups reporting high levels of confidence in completing medical forms independently.

Most mothers in both groups disagreed that their child was different from most people (VUS cases: 76% (32/42), controls: 81% (66/81)). Similarly, 72% (31/42) of mothers in the VUS group and 80% (65/81) of control mothers disagreed that their child’s genetic makeup made them different. No significant differences were found between the groups on these measures.

### CNV reanalysis

After excluding 3 participants who withdrew from the study, 56 mothers consented to having their child’s prenatal CNV result reanalyzed. An average period of 5.5 years had elapsed since the original prenatal microarray report.

Of the 47 participants with a prenatal VUS, 31 (66%) were downgraded to “no clinically significant genomic imbalance detected,” 4 (9%) were upgraded to “pathogenic copy-number change with variable expressivity,” and 12 (26%) remained unchanged as a VUS. Table 3 provides the details of the reanalysis of VUS. The majority of VUS were inherited variants, accounting for 72% (34/47), whereas 23% (11/47) were undetermined (meaning that the parents were not tested; therefore, this could not be evaluated), and 4% (2/47) were de novo variants (see Table 3).

**Table 3:** Reanalysis of copy-number variants of uncertain significance (VUS *N* = 47)^a^

ID	Year of Prenatal Report	Molecular Karyotype Result	Result of CNV Reanalysis	Updated CNV Classification
1	2013	arr[hg19] 1p22.1(92,091,953-92,537,856)x3 mat	Downgrade	No clinically significant genomic imbalance detected
2	2014	arr[hg19] 2p11.2(86,298,862-86,480,995)x3 pat	Downgrade	No clinically significant genomic imbalance detected
3	2014	arr[hg19] 1q21.1(145,382,123-145,988,238)x3 pat	Downgrade	No clinically significant genomic imbalance detected
4	2014	arr[hg19] 2p21(44,573,290-44,714,907)x1,2p16.3p16.2(52,601,319-54,007,183)x3	Downgrade	No clinically significant genomic imbalance detected
5	2014	arr[hg19] 8q13.2(68,111,228-68,336,194)x3 pat	Downgrade	No clinically significant genomic imbalance detected
6	2014	arr[hg19] 20q13.33(61,513,870-62,325,333)x3 pat	Downgrade	No clinically significant genomic imbalance detected
7	2014	arr[hg19] 15q25.2q25.3(84,952,634-85,714,129)x3	Downgrade	No clinically significant genomic imbalance detected
8	2015	arr[hg19] 7q31.2q31.31(117,387,108-118,978,523)x3	Downgrade	No clinically significant genomic imbalance detected
9	2015	arr[hg19] 3q13.31(113,862,887-114,208,597)x3 mat	Downgrade	No clinically significant genomic imbalance detected
10	2015	arr[hg19] 11q24.3(128,620,132-128,843,852)x3	Downgrade	No clinically significant genomic imbalance detected
11	2015	arr[hg19] 15q11.2(22,754,322-23,222,284)x1 pat	Downgrade	No clinically significant genomic imbalance detected
12	2016	arr[hg19] 7p15.2(27,135,314-27,398,564)x3 mat	Downgrade	No clinically significant genomic imbalance detected
13	2016	arr[hg19] 2q23.3(152,775,308-153,346,845)x3	Downgrade	No clinically significant genomic imbalance detected
14	2016	arr[hg19] 3q26.31(173,868,469-174,434,433)x3 pat	Downgrade	No clinically significant genomic imbalance detected
15	2017	arr[hg19] 1p31.1(79,120,788-81,873,869)x3mat	Downgrade	No clinically significant genomic imbalance detected
16	2017	arr[hg19] 18q21.2(53,167,520-53,676,203)x3 mat	Downgrade	No clinically significant genomic imbalance detected
17	2017	arr[hg19] Xp11.22(50,424,688-50,659,572)x2 mat	Downgrade	No clinically significant genomic imbalance detected
18	2017	arr[hg19] 19p13.2(10,031,745-10,230,531)x3 mat	Downgrade	No clinically significant genomic imbalance detected
19	2017	arr[hg19] 15q13.3(32037769-32615660)x3 pat	Downgrade	No clinically significant genomic imbalance detected
20	2017	arr[hg19] 2q23.3q24.1(154,808,697-155,234,318)x1 pat	Downgrade	No clinically significant genomic imbalance detected
21	2017	arr[hg19] 5q13.2(70,307,162-70,308,607)x0	Downgrade	No clinically significant genomic imbalance detected
22	2017	arr[hg19] 16p13.11p12.3(15,491,445-18,196,549)x3 dn	Downgrade	No clinically significant genomic imbalance detected
23	2018	arr[hg19] 15q11.2(22,772,887-23,226,254)x1 pat	Downgrade	No clinically significant genomic imbalance detected
24	2018	arr[hg19] 22q11.21(20,225,536-21,247,007)x3	Downgrade	No clinically significant genomic imbalance detected
25	2018	arr[hg19] 3p26.1p25.3(6,872,696-9,534,149)x3 pat	Downgrade	No clinically significant genomic imbalance detected
26	2018	arr[hg19] 19p13.3(2,833,431-3,179,517)x1	Downgrade	No clinically significant genomic imbalance detected
27	2018	arr[hg19] 21q22.3(46,427,271-47,409,043)x3 mat	Downgrade	No clinically significant genomic imbalance detected
28	2018	arr[hg19] 8q23.3q24.12(117,296,350-119,453,249)x3 mat	Downgrade	No clinically significant genomic imbalance detected
29	2018	arr[hg19] 1p36.32(3252104-4405998)x3	Downgrade	No clinically significant genomic imbalance detected
30	2019	arr[hg19] 4q35.2(188345346-190937862)x1 mat	Downgrade	No clinically significant genomic imbalance detected
31	2019	arr[GRCh37] 2q23.3(152720030-153169807)x3 pat	Downgrade	No clinically significant genomic imbalance detected
32	2013	arr[hg19] 10q22.3(79,167,302-79,569,505)x1	Unchanged	Copy number change of unknown clinical significance
33	2014	arr[hg19] 17p13.3(1,290,619-1,591,699)x3 pat	Unchanged	Copy number change of uncertain significance
34	2014	arr[hg19] 4p16.1(9,690,750-10,451,450)x3 pat	Unchanged	Copy number change of uncertain significance
35	2015	arr[hg19] Xp22.13(18,550,565-18,928,110)x3 mat	Unchanged	Copy number change of uncertain significance
36	2016	arr[hg19] 16p12.2(21,966,869-22,392,905)x1 mat	Unchanged	Copy number change of uncertain significance
37	2017	arr[hg19] 4q34.3(178,181,793-179,856,502)x3	Unchanged	Copy number change of uncertain significance
38	2017	arr[hg19] Xp22.31(6039361-6413964)x3 mat	Unchanged	Copy number change of uncertain significance
39	2018	arr[hg19] 15q11.2(22318597-23089190)x1 pat	Unchanged	familial chromosome imbalance of unknown clinical significance detected
40	2018	arr[hg19] 1p36.33(835,092-1,964,852)x3 mat	Unchanged	Copy number change of unknown significance
41	2018	arr[hg19] 20p12.2p12.1(11,157,244-15,421,770)x3 pat	Unchanged	Copy number change of unknown significance
42	2019	arr[hg19] 9q33.1(119185778-119476270)x1 dn	Unchanged	Copy number change of unknown significance
43	2019	arr[hg19] 4p16.2(4812152-5831292)x3 mat	Unchanged	Copy number change of uncertain significance
44	2016	arr[hg19] 16p11.2(29,595,483-30,198,151)x?2∼3	Upgrade	Pathogenic number variant with variable expressivity
45	2018	arr[hg19] 1q21.1q21.2(146506339-147824177)x1 pat	Upgrade	Pathogenic copy number change with variable expressivity
46	2018	arr[GRCh37] 2q11.1q11.2(96766589-98019615)x1 pat	Upgrade	Pathogenic copy number change
47	2018	arr[hg19] 1q21.1q21.2(146506339-147824177)x3 mat	Upgrade	Pathogenic copy number change with variable expressivity

*CNV*, copy-number variant; *dn*, de novo; *mat*, maternal; *pat*, paternal; *und*, undetermined; *VUS*, variant of uncertain significance.

a Note: VUS *N* = 47 for CNV reanalysis because 1 participant consented to prenatal microarray reanalysis but did not complete the questionnaire or clinical outcome measures.

We conducted a post hoc analysis comparing clinical assessment outcomes for children whose VUS results are no longer reported (*n* = 19) with the controls (*n* = 50) and found no significant differences in intellectual functioning, adaptive functioning, or social communication skills associated with ASD (see Supplemental Table 7). Similarly, when comparing these outcomes for children whose VUS results are still reported or have been reclassified as pathogenic with variable expressivity (*n* = 11) with the controls (*n* = 50), no differences were observed (see Supplemental Table 8).

## Discussion

We conducted a comprehensive evaluation of the developmental, social-emotional, and health outcomes of children with and without a prenatal diagnosis of a VUS at a mean age of 6 years. The results are reassuring, demonstrating that children with a VUS performed similarly to children without a VUS across a wide range of developmental, cognitive, and health measures. Our study found no significant differences in maternal perceptions of child health, behavior, or development between the VUS and control groups. A key concern with genomic testing and disclosure of uncertain findings is the potential for increased anxiety, which may negatively affect parenting styles and the mother-child relationship.^40^ However, mothers in our VUS and control groups reported similar levels of mental health, parental competence, and family functioning. Taken together, these findings suggest no enduring impacts through early childhood of a prenatal diagnosis of a VUS on either child health and development or aspects of family well-being.

Our research makes a major contribution to the limited evidence base for this population. Only 2 other studies have followed up large cohorts with a prenatal diagnosis of a CNV. The Belgian prenatal microarray cohort study used the Ages and Stages Questionnaire to follow up children who had a prenatal diagnosis of a CNV (85 cases, 123 controls). Compared with controls, they identified significant delays in communication and personal-social development among 3-year-olds with a susceptibility CNV. However, this study specifically excluded children with a VUS from further analyses, limiting any direct comparisons with this study.^41^ More recently, Shi et al^42^ followed 113 infants with a prenatal VUS, conducting follow-up at 2 time points: 0 to 10 months and 2 to 4 years of age. They reported 5 with potential disease phenotypes, but the clinical assessments were not described, participant attrition rate was high, and there was no comparator group, thus precluding any firm conclusions about the childhood outcomes for this sample.

In contrast, our cohort has been assessed with a broad range of structured, validated tools involving a variety of data sources: in-person assessment of cognitive function by a psychologist, clinical assessment by a pediatrician, and parent-reported measures of adaptive behavior and function. Our study also has the longest follow-up period, with an average age of approximately 6 years, compared with 3 years in the Belgium microarray cohort^41^ and 4 years in Shi et al.^42^ Importantly, our inclusion of a control group mitigated potential pandemic-related factors affecting measured outcomes.

We intentionally used many of the same parent-rated measures as Desai et al,^43^ who investigated parental perceptions of their children after a prenatal diagnosis of a VUS. Desai et al^43^ found that parents of VUS children expressed greater concerns about their child’s competence at 12 months, although these concerns resolved by 36 months. Our findings at an average age of 6 years build on their results, providing no evidence of long-term concerns into early childhood. Desai et al^43^ also reported that parents of VUS children had lower satisfaction with their decision to undergo prenatal testing compared with the control group.^43^ Although our study observed a similar trend, the group differences were not statistically significant and average decisional satisfaction scores were high (>40/50) across both groups in both studies.

Consistent with our reassuring findings, the blinded reanalysis conducted by the original reporting laboratories indicated that two-thirds of the children in the VUS group would be now classified as having “no clinically significant genomic imbalance detected.” This corresponds with advances in knowledge and updated reporting practices^6^ and an observed decline in VUS results recorded in the Victorian Prenatal Diagnosis Data collection.^17^ Over the past decade in the Australian state of Victoria, the annual number of fetuses identified with a variant of uncertain significance (VUS) has declined from 97 cases (4% of prenatal diagnostic tests) in 2013 to 18 cases (1%) in 2022.^17^ This dramatic decline is due to both a reduction in total prenatal diagnostic procedures (due to the increasing use of prenatal cell-free DNA screening) and a reduction in the rate of VUS reported per CMA.

The 75% VUS reclassification rate observed in our study (66% downgraded, 9% upgraded) highlights the clinical value of structured reanalysis, particularly when the updated classification offers reassurance to families or guides future care. However, reclassification rates and the proportions of upgraded versus downgraded results vary across studies and are shaped by multiple factors. In a China-based cohort, Shi et al^42^ reanalyzed 139 VUS originally classified using the 2015 ACMG guidelines,^44^ applying the updated 2019 standards.^6^ They reported a reclassification rate of 17%, with 11% downgraded and 7% upgraded. In a US study, Drackley et al^45^ reclassified 145 difficult-to-classify CNVs using both an in-house laboratory system and the 2019 ACMG guidelines, identifying a 39.3% discordance rate (*n* = 57). Of these 145 CNVs, 33% were upgraded from benign to VUS, 3% downgraded from pathogenic to VUS, and 3% upgraded from VUS to pathogenic—resulting in an overall upgrade rate of 36%. These differences highlight how evolving guidelines and laboratory practices—including reporting thresholds, phenotype use, and database selection—can influence how VUS are reclassified. This contextual variability is important to consider when interpreting and comparing rates across studies.

Although VUS reclassification offers clear benefits, it also presents ethical, clinical, and logistical challenges, particularly when variants are upgraded. Similar issues arise in fetal exome sequencing, where automated reanalysis is being promoted.^46^, ^47^, ^48^ Qualitative research to understand the parental experience of a reclassified prenatal VUS is currently underway among our PALM cohort and will be reported separately in a future publication. The high rate of VUS reclassification to “likely benign” supports a conservative reporting approach in the prenatal setting, given the potential for psychological distress in pregnancy and absence of detectable developmental differences in early childhood.

Creating pediatric cohorts from the prenatal diagnostic testing population is valuable for understanding long-term outcomes of genomic information gained before birth. However, there were logistical challenges in this study and multiple unavoidable potential sources of bias. First, our inclusion of only liveborn outcomes likely excluded cases of VUS that contributed to fetal demise or were associated with severe ultrasound abnormalities that prompted termination of pregnancy. Second, the requirement for English proficiency likely excluded families from lower socioeconomic status or culturally diverse backgrounds, introducing further bias. Third, our recruitment was only 21% among successfully contacted families. This rate is comparable to other international studies but still potentially contributes to selection bias. We previously reported in detail on the screening process and attrition^18^ and noted that among controls, responders tended to have higher socioeconomic status and lower parity than nonresponders. However, there were no differences in metropolitan/rural status, maternal age, or child’s age at recruitment between responders and nonresponders within the control group. No differences in sociodemographic factors were noted between responders and nonresponders in the VUS group.^18^ Moreover, our previous work has shown that pregnancies with inherited VUS or without ultrasound abnormalities were more likely to proceed to live birth, which may have influenced the composition of the cohort.^49^ Although we used standardized developmental assessments with population-based norms and adjusted for multiple demographic variables, residual confounding cannot be ruled out. These factors should be considered when interpreting the generalizability of our findings.

Our recruitment rate of 21%, although modest, compares favorably with similar international cohorts, such as the Belgian microarray cohort, which had a recruitment rate of 17%.^41^ Given that our study imposed a higher participation burden—requiring in-person child assessments in addition to longer parent questionnaires—we considered the recruitment rate acceptable in the context of the societal disruptions of the COVID-19 pandemic.^50^ In total, 29 mother-child pairs with prenatal VUS completed all survey and clinical assessments. Although this limits our ability to detect subtle differences, the mean FSIQ scores for both groups were well within the age-normed average range (25th-75th percentiles). Larger cohorts are needed to confirm these findings. We await the results of the US microarray follow-up study by Wapner et al^2^ (Clinical Trials #NCT02160938), which has used the same developmental instruments (WPPSI-IV, WISC-V, and Vineland-3), allowing for future comparative analyses or meta-analysis.

### Conclusion

Children born after a prenatal diagnosis of a VUS have similar intellectual functioning, adaptive living skills, and social behaviors compared with those without a VUS. Likewise, their mothers report comparable levels of mental health, parental competence, and family functioning to controls. Collectively, these findings suggest no lasting impact of a prenatal VUS diagnosis on either child development or family well-being. This contributes valuable evidence to support prenatal genetic counseling and clinical laboratory reporting practices.

## Data Availability

The data that support the findings of this study are available from the corresponding author to researchers from a recognized academic institution upon request.

## ORCID

Lisa Hui: https://orcid.org/0000-0002-9720-3562

## Conflict of Interest

The authors declare no conflicts of interest.
