# Supplementary material for: Childhood outcomes of fetal genomic copy-number variants: The prenatal microarray cohort study
Source: Genet Med Open. 2025 Oct 13;3:103464. doi: 10.1016/j.gimo.2025.103464 (PMC12681968; doi:10.1016/j.gimo.2025.103464)
Supplement: Supplementary Methods [file mmc1.pdf]

Supplementary methods

Annotation software used by Victorian Clinical Genetics Services (VCGS) and Monash Pathology for the CNV reanalysis.

Reanalysis of copy number variants was performed on enrolled participants with VUS and clinically significant CNVs using the clinical standard laboratory software and protocols in use during 2022-23. This included use of Biodiscovery NxClinical (v5.1) and Bionano VIA (v7.1) analysis software, that allowed for the visualisation and pre-classification of CNVs based on comparison of the identified CNV with other CNVs identified by the laboratory, RefSeq and OMIM gene content, the prevalence in the Database of Genomic Variation (DGV) database, and ClinGen Haploinsufficiency/ Triplosensitivity scores [https://www.clinicalgenome.org/curation-activities/dosage-sensitivity/]. The CNVs were classified in accordance with the ACMG (American College of Medical Genetics and Genomics) CNV technical standard (1).

We compared the classifications given at the time of issue of the prenatal report with the current classifications in 2022-23, according to the issuing laboratory's protocols. The proportions of pCNVs, VUS, and benign variants in the enrolled cohort were compared between the prenatal and postnatal classifications using a chi-squared test for proportions.

1. Riggs ER, Andersen EF, Cherry AM, Kantarci S, Kearney H, Patel A, et al. Technical standards for the interpretation and reporting of constitutional copy-number variants: a joint consensus recommendation of the American College of Medical Genetics and Genomics (ACMG) and the Clinical Genome Resource (ClinGen). Genet Med. 2020;22(2):245-57.

Refer to the excel file for Supplementary Table 1. Description of baseline questionnaires

Refer to the excel file for Supplementary Table 2. Perinatal outcomes

Supplementary Table 3. VUS cases with an ultrasound abnormality reported prior to prenatal diagnostic testing (N=32)[a]

ID	Gestation at prenatal diagnosis (weeks)	Ultrasound indication for prenatal diagnosis
1	21	Absent nasal bone
2	31	Mild unilateral cerebral ventriculomegaly (12mm)
3	23	Hypoplastic nasal bone
4	13	Subcutaneous edema, nuchal translucency (3.2mm)
5	21	Agenesis of the corpus callosum
6	34	Right pleural effusion
7	22	Right sided aortic arch
8	21	Unilateral mild cerebral ventriculomegaly (11.5m)
9	21	Ventriculoseptal defect, suspected horseshoe kidney
10	21	Pulmonary stenosis
11	24	Ventriculoseptal defect, fetal growth restriction
12	23	Bilateral mild cerebral ventriculomegaly
13	24	Unilateral multicystic dysplastic kidney
14	23	Aberrant right subclavian artery
15	25	Atrioventricular septal defected
16	21	Unilateral cleft lip and palate, single umbilical artery, ambiguous genitalia
17	23	Unilateral cleft lip and palate
18	20	Bilateral talipes
19	16	Increased nuchal translucency (4.5mm), absent nasal bone
20	16	Increased nuchal translucency (4.2mm), dilated right jugular sac
21	16	Increased nuchal translucency (4.3mm)
22	22	Right sided aortic arch
23	15	Increased nuchal translucency
24	16	Increased nuchal translucency (3.1mm)
25	15	Increased nuchal translucency (4.2mm), dilated right jugular sac
26	22	Increased nuchal fold (8mm)
27	22	Cleft lip and palate
28	13	Increased nuchal translucency with dilated jugular sacs
29	14	Increased nuchal translucency (2.9mm)
30	15	Increased nuchal translucency (6.2mm)
31	15	Increased nuchal translucency (3.2mm)
32	15	Increased nuchal translucency (3.2mm)

[a] Note: *n*=32 comprised those with a structural anomaly (*n*=26), increased nuchal translucency measurement as part of a positive combined first trimester screening result (*n*=5) and cleft lip and palate with a positive second trimester serum screening result (*n*=1) (as shown in **Supplementary Table 2).**

Supplementary Table 4. Comparison of children with and without a VUS on measures of intellectual functioning, adaptive functioning, and behaviours associated with autism spectrum disorder, unadjusted and adjusted regression analysis results

**Measure** (Population Mean (SD))	**Domain** (Higher scores indicate higher levels of the domain measured)	**VUS (n=30)**[a] Median (IQR)/Mean(SD)	**Control (n=50)**[b] Median (IQR)/Mean(SD)	**Unadjusted**		**Adjusted**[e]	
				**β (95% CI)**	**p**	**β (95% CI)**	**p**
**WPPSI-IV / WISC-V** (100 (15))	Overall intellectual functioning	96.3 (14.1)	101.5 (16.0)	-0.005 (-0.012, 0.002)	0.14	-0.003 (-0.010, 0.005)	0.52
	Working memory	98.4 (16.6)	101.2 (15.8)	-0.003 (-0.009, 0.004)	0.47	0.000 (-0.008, 0.007)	0.95
	Visual-spatial reasoning	99.8 (14.6)	101.2 (14.9)	0.000 (-0.008, 0.007)	0.95	0.001 (-0.007, 0.009)	0.82
	Verbal comprehension	95.5 (13.8)	103.7 (14.6)	-0.009 (-0.016, -0.002)	0.02	-0.007 (-0.015, 0.001)	0.08
	Fluid reasoning	96.6 (17.7)	97.9 (15.6)	-0.001 (-0.009, 0.006)	0.75	0.001 (-0.007, 0.009)	0.74
	Processing speed	97.2 (13.5)	99.8 (14.8)	-0.003 (-0.012, 0.006)	0.49	-0.003 (-0.012, 0.007)	0.58
**Vineland-3 Parent/Caregiver form** (100 (15))	Overall adaptive functioning	93.1 (13.9)	92.2 (10.5)	0.002 (-0.008, 0.011)	0.73	0.002 (-0.008, 0.011)	0.74
	Communication	94.5 (82,102)	94.5 (88,100)	0.000 (-0.009, 0.010)	0.93	0.002 (-0.008, 0.012)	0.70
	Daily Living Skills	93.7 (13.7)	92.9 (13.3)	0.001 (-0.007, 0.009)	0.78	0.001 (-0.008, 0.009)	0.91
	Socialization	96.1 (13.9)	95.9 (10.1)	0.000 (-0.009, 0.010)	0.94	0.001 (-0.009, 0.010)	0.87
	Motor skills (gross and fine)	96 (89, 102)	96 (89,104)	-0.001 (-0.010, 0.007)	0.74	-0.002 (-0.011, 0.007)	0.67
**Vineland-3 Parent/ Caregiver form** Maladaptive scales[c]	Internalizing behaviours (e.g., anxiety)	17 (15,18)	15 (14,18)	0.011 (-0.028, 0.051)	0.57	0.009 (-0.028, 0.046)	0.63
	External behaviours (e.g., outbursts)	17 (14,19)	17 (14,18)	0.014 (-0.027, 0.055)	0.49	0.002 (-0.039, 0.044)	0.91
**SRS-2** (T-scores)[d]	Overall score of behaviours associated with autism spectrum disorder	51.5 (46,59.5)	49 (43.5,55.5)	0.008 (-0.002, 0.018)	0.13	0.005 (-0.006, 0.017)	0.34
	DSM-5 Compatible Scale: Social Communication and Interaction	52 (44.5,61)	48.5 (43.5,55.5)	0.006 (-0.004, 0.017)	0.23	0.005 (-0.006, 0.015)	0.38
	DSM-5 Compatible Scale: Restricted Interests and Repetitive Behavior	48 (45.5, 59.5)	47 (43,55)	0.004 (-0.006, 0.013)	0.46	0.001 (-0.009, 0.011)	0.86

[a] The VUS sample size for each outcome ranged between n= 24 - 30.
[b] The control sample size for each outcome ranged between n= 39 - 50.
[c] Maladaptive behaviours (Vineland-3)* v-Scale score 0-24 range; Mean = 15; SD=3
[d] ≤59 = within normal limits (generally not associated with autism spectrum disorder); between 60-65 = mild range; between 66-75 = moderate range; ≥76 = severe range (strongly associated with clinical diagnosis of autism spectrum disorder)
[e] Maternal age at child's birth, maternal country of birth, maternal education level, maternal relationship status, income, and locality (metro/regional)

Supplementary Table 5. Clinical concerns on paediatric review

	Controls (n=49)		VUS cases (n=29)		p
	n	%	n	%	
**Developmental domains**					
**Growth**	0	0%	2	7%	0.06
**Development**	13	27%	10	35%	0.46
**Cognitive**	4	8%	6	21%	0.11
**Behavioural**	16	33%	10	34%	0.87
**Neurological**	2	4%	3	10%	0.28
**Medical domains**					
**Blood**	2	4%	3	10%	0.28
**Cardiac**	2	4%	5	17%	0.05
**Dermatological**	28	57%	14	48%	0.45
**Dietary**	9	18%	6	21%	0.80
**Endocrine**	0	0%	2	7%	0.06
**Gastrointestinal**	10	20%	7	24%	0.70
**Hearing**	2	4%	7	24%	0.01
**Infection**	6	12%	11	38%	0.01
**Liver**	0	0%	1	3%	0.19
**Ocular**	10	20%	2	7%	0.11
**Orthopaedic**	8	16%	3	10%	0.46
**Pulmonary**	8	16%	9	31%	0.13
**Renal**	6	12%	5	17%	0.54
**Dysmorphic**					
Yes	2	4%	5	17%	0.12
No	44	92%	22	76%	
Possibly	2	4%	2	7%	

Supplementary Table 6. Comparison of mothers of children with and without a VUS on measures of maternal mental health and parenting and perceptions of their child's behaviour and development

**Measure** (Score range)	**Domain** (Higher scores indicate higher levels of the domain measured)	**Mother of child with a VUS (N=46)[a]** Median (IQR)/Mean(SD)	**Mother of child in Control group (N=88)[b]** Median (IQR)/Mean(SD)	**p**
PHQ8 (0 – 24)	Depressive symptoms	2.0 (0.0, 5.0)	2.0 (1.0, 6.0)	0.35
STAI-S (0 – 80)	Short-term anxiety (state)	27.5 (21.5, 37.5)	34.0 (26.0, 44.0)	0.08
STAI-T (0 – 80)	Long-term anxiety (trait)	34.0 (27.0, 42.0)	36.0 (28.0, 47.0)	0.20
PSOC (0 – 102)	Parental competency levels	76.0 (63.0, 87.0)	71.0 (62.0, 81.0)	0.38
VCS (16 – 64)	Child vulnerability[d]	55.5 (50.0, 59.0)	58.0 (50.0, 60.0)	0.10
DSS (10 – 50)	Satisfaction about having a prenatal microarray	42.0 (39.0, 44.0)	44.0 (40.0, 48.0)	0.02
SDQ (z-scores)[c]	Total score	0.1 (0.1, 0.8)	-0.4 (-0.9, 0.5)	0.07
SDQ subscales (z-scores)[c]	Emotional problems	0.4 (-0.6, 0.8)	-0.3 (-0.9, 0.4)	0.04
	Conduct problems	-0.2 (-0.9, 0.8)	-0.3 (-0.9, 0.3)	0.26
	Hyperactivity	0.0 (-0.8, 0.7)	-0.2 (-0.8, 0.3)	0.23
	Peer problems	-0.2 (-0.8, 0.7)	-0.6 (-0.8, 0.4)	0.21
	Prosocial behaviours	0.0 (-0.5, 0.5)	0.5 (-0.5, 1.0)	0.15
MFFS (0 - 48)	Family functioning	20.0 (15.5, 24.0)	18.0 (14.0, 23.0)	0.36
RSAT (0 - 20)	Uncertainty tolerance	10.3 (3.4)	10.6 (3.2)	0.66
GKS (0 - 25)	Genetic knowledge	17.5 (13.0, 21.0)	18.0 (14.0, 21.5)	0.29

[a] The VUS sample size for each outcome ranged between n= 40 - 46.

[b] The Control sample size for each outcome ranged between n= 80 - 88.

[c] A z-score is the number of standard deviations a given data point (X) is from the average and can be positive (+) or negative (-) in value. A z-score of 1 means the data point is 1 standard deviation above the average; a z-score of -1 means the point is 1 standard deviation below the average.

[d] Higher scores indicate lower levels of child vulnerability.

Supplementary Table 7. Measures of intellectual functioning, adaptive functioning, and behaviours associated with autism spectrum disorder between children with a no longer reported VUS and children without a VUS

**Measure** (Population Mean (SD))	**Domain** (Higher scores indicate higher levels of the domain measured)	**VUS (n=19)**[a] Median (IQR)/Mean(SD)	**Control (n=50)**[b] Median (IQR)/Mean(SD)	**p**
**WPPSI-IV / WISC-V** (100 (15))	Overall intellectual functioning	95.9 (14.7)	101.5 (16.0)	0.20
	Working memory	99.1 (15.7)	101.2 (15.8)	0.64
	Visual-spatial reasoning	102.3 (13.8)	101.2 (14.9)	0.79
	Verbal comprehension	94.7 (15.9)	103.7 (14.6)	0.03
	Fluid reasoning	94.8 (18.8)	97.9 (15.6)	0.52
	Processing speed	100.6 (13.6)	99.8 (14.8)	0.85
**Vineland-3 Parent/Caregiver form** (100 (15))	Overall adaptive functioning	93.9 (12.2)	92.2 (10.5)	0.56
	Communication	96.0 (88.0, 102.0)	94.5 (88.0, 100.0)	0.68
	Daily Living Skills	95.6 (11.4)	92.9 (13.3)	0.43
	Socialization	96.3 (14.1)	95.9 (10.1)	0.91
	Motor skills (gross and fine)	96.0 (~)	96.0 (89.0, 104.0)	0.97
**Vineland-3 Parent/Caregiver form** Maladaptive scales[c]	Internalizing behaviours (e.g., anxiety)	16.0 (14.0, 18.0)	15.0 (14.0, 18.0)	0.97
	External behaviours (e.g., outbursts)	18.0 (14.0, 19.0)	17.0 (14.0, 18.0)	0.53
**SRS-2** (T-scores)[d]	Overall score of behaviours associated with Autism Spectrum Disorder	49.5 (46.0, 59.0)	49.0 (43.5, 54.4)	0.38
	DSM-5 Compatible Scale: Social Communication and Interaction	48.0 (44.5, 61.0)	48.5 (43.5, 55.5)	0.74
	DSM-5 Compatible Scale: Restricted Interests and Repetitive Behavior	48.0 (46.0, 57.5)	47.0 (43.0, 55.0)	0.26

[a] The VUS sample size for each outcome ranged between n= 16 - 19.

[b] The Control sample size for each outcome ranged between n= 39 - 50.

[c] Maladaptive behaviours (Vineland-3)* v-Scale score 0-24 range; Mean = 15; SD=3

[d] ≤59 = within normal limits (generally not associated with autism spectrum disorder); between 60-65 = mild range; between 66-75 = moderate range; ≥76 = severe range (strongly associated with clinical diagnosis of autism spectrum disorder)

Supplementary Table 8. Measures of intellectual functioning, adaptive functioning, and behaviours associated with autism spectrum disorder between children with a still reported/upgrade VUS and children without a VUS

**Measure** (Population Mean (SD))	**Domain** (Higher scores indicate higher levels of the domain measured)	**VUS (n=11)**[a] Median (IQR)/Mean(SD)	**Control (n=50)**[b] Median (IQR)/Mean(SD)	**p**
**WPPSI-IV / WISC-V** (100 (15))	Overall intellectual functioning	96.1 (14.0)	101.5 (16.0)	0.30
	Working memory	96.6 (19.1)	101.2 (15.8)	0.41
	Visual-spatial reasoning	94.5 (14.9)	101.2 (14.9)	0.18
	Verbal comprehension	95.1 (9.3)	103.7 (14.6)	0.07
	Fluid reasoning	99.9 (17.1)	97.9 (15.6)	0.76
	Processing speed	91.9 (12.4)	99.8 (14.8)	0.17
**Vineland-3 Parent/Caregiver form** (100 (15))	Overall adaptive functioning	91.8 (16.9)	92.2 (10.5)	0.92
	Communication	88.0 (81.0, 102.0)	94.5 (88.0, 100.0)	0.67
	Daily Living Skills	90.5 (17.1)	92.9 (13.3)	0.61
	Socialization	95.9 (14.1)	95.9 (10.1)	0.99
	Motor skills (gross and fine)	96.0 (92.0, 100.0)	96.0 (89.0, 104.0)	0.83
**Vineland-3 Parent/Caregiver form** Maladaptive scales[c]	Internalizing behaviours (e.g., anxiety)	17.5 (15.0, 20.0)	15.0 (14.0, 18.0)	0.35
	External behaviours (e.g., outbursts)	16.0 (14.0, 19.0)	17.0 (14.0, 18.0)	0.49
**SRS-2** (T-scores)[d]	Overall score of behaviours associated with Autism Spectrum Disorder	52.0 (47.0, 63.0)	49.0 (43.5, 54.4)	0.23
	DSM-5 Compatible Scale: Social Communication and Interaction	53.0 (48.0, 63.5)	48.5 (43.5, 55.5)	0.19
	DSM-5 Compatible Scale: Restricted Interests and Repetitive Behavior	48.0 (43.5, 61.0)	47.0 (43.0, 55.0)	0.59

[a] The VUS sample size for each outcome ranged between n= 8 - 11.

[b] The Control sample size for each outcome ranged between n= 39 - 50.

[c] Maladaptive behaviours (Vineland-3)* v-Scale score 0-24 range; Mean = 15; SD=3

[d] ≤59 = within normal limits (generally not associated with autism spectrum disorder); between 60-65 = mild range; between 66-75 = moderate range; ≥76 = severe range (strongly associated with clinical diagnosis of autism spectrum disorder)
