# Supplementary material for: Childhood outcomes of fetal genomic copy-number variants: The prenatal microarray cohort study
Source: Genet Med Open. 2025 Oct 13;3:103464. doi: 10.1016/j.gimo.2025.103464 (PMC12681968; doi:10.1016/j.gimo.2025.103464)
Supplement: Supplementary Data [file mmc2.docx]

**Supplementary methods**

Annotation software used by Clinical Service and Laboratory 1 and Clinical Service and Laboratory 2 for the CNV reanalysis.

Reanalysis was performed on enrolled participants with VUS and clinically significant CNVs using the clinical standard laboratory software and protocols in use during 2022-23. This included use of Biodiscovery NxClinical (v5.1) and Bionano VIA (v7.1) analysis software, that allowed for the visualisation and pre-classification of CNVs based on comparison of the identified CNV with other CNVs identified by the laboratory, RefSeq and OMIM gene content, the prevalence in the Database of Genomic Variation (DGV) database, and ClinGen Haploinsufficiency/ Triplosensitivity scores [https://www.clinicalgenome.org/curation-activities/dosage-sensitivity/]. The CNVs were classified in accordance with the ACMG (American College of Medical Genetics and Genomics) CNV technical standard (1).

We compared the classifications given at the time of issue of the prenatal report with the current classifications in 2022-23, according to the issuing laboratory’s protocols. The proportions of pCNVs, VUS, and benign variants in the enrolled cohort were compared between the prenatal and postnatal classifications using a chi-squared test for proportions.

1. Riggs ER, Andersen EF, Cherry AM, Kantarci S, Kearney H, Patel A, et al. Technical standards for the interpretation and reporting of constitutional copy-number variants: a joint consensus recommendation of the American College of Medical Genetics and Genomics (ACMG) and the Clinical Genome Resource (ClinGen). Genet Med. 2020;22(2):245-57.

| **Supplementary Table 1. Description of baseline questionnaires** | | |
| --- | --- | --- |
| **Questionnaire** | **Description** | **Reference** |
| Patient Health Questionnaire (PHQ) | The Patient Health Questionnaire is an 8-item self-report questionnaire used for screening and measuring depressive symptoms over the last 2 weeks. Items are scored on a 4-point scale ranging from 0 ("not at all") to 3 ("nearly every day"). The total score ranges from 0 to 24, with higher scores indicating more severe depressive symptoms. The scoring thresholds for interpreting the severity of depression are the same as those of the PHQ-9: 0-4 (minimal or no depression), 5-9 (mild depression), 10-14 (moderate depression), 15-19 (moderately severe depression), and 20-24 (severe depression). | Kroenke K, Spitzer RL, Williams JBW. The PHQ-9: validity of a brief depression severity measure. Journal of General Internal Medicine. 2001;16(9):606. |
| State Trait Anxiety Inventory  (STAI-S &  STAI-T) | The State-Trait Anxiety Inventory (STAI) is a widely used self-report instrument designed to measure two types of anxiety: state anxiety (STAI-S) and trait anxiety (STAI-T). The STAI consists of 40 items, with 20 items assessing state anxiety and 20 items assessing trait anxiety. Respondents rate how they feel "right now, at this moment" for state anxiety and how they "generally feel" for trait anxiety, using a 4-point Likert scale ranging from 1 ("not at all") to 4 ("very much so"). The total score for each subscale ranges from 20 to 80, with higher scores indicating greater anxiety levels. A cut-off score of 40 is commonly used to define probable clinical levels of anxiety. | Spielberger CD, Gorsuch RL, Lushene R, Vagg PR, Jacobs GA. *Manual for the State-Trait Anxiety Inventory (STAI)*. Consulting Psychologists Press; 1983. |
| Parenting Sense of Competence Scale (PSOC)^a^ | The Parenting Sense of Competence Scale (PSOC) is a self-report measure designed to assess parents' perceptions of their competence in the parenting role. The PSOC consists of 16 items, divided into two subscales: Satisfaction and Efficacy. The Satisfaction subscale measures a parent's satisfaction with their parenting role, while the Efficacy subscale assesses a parent's perception of their effectiveness and competence in parenting tasks. Responses are rated on a 6-point Likert scale, ranging from 1 ("strongly disagree") to 6 ("strongly agree"). Higher scores on the PSOC indicate a greater sense of competence in parenting. | Gibaud‐Wallston, J., & Wandersman, L. P. (1978). Parenting  sense of competence scale. Lawrence Erlbaum Associates.  Chicago |
| McMaster Family Functioning Subscale (MFFS) | The McMaster Family Functioning Subscale (FAD-GF) is a self-report measure designed to assess a family unit's overall health and functioning. It is part of the larger McMaster Family Assessment Device (FAD) and specifically evaluates general family functioning. The subscale consists of 12 items, which respondents rate on a 4-point Likert scale ranging from 1 ("strongly agree") to 4 ("strongly disagree"). The FAD-GF assesses various aspects of family life, including communication, problem-solving, emotional involvement, and behaviour control. Higher scores on the subscale indicate poorer family functioning, while lower scores suggest healthier family dynamics. | Epstein NB, Baldwin LM, Bishop DS. The McMaster Family Assessment Device. *Journal of Marital and Family Therapy*. 1983;9(2):171-180. |
| Revised Scale for Ambiguity Tolerance (RSAT)^a^ | The Revised Scale for Ambiguity Tolerance (RSAT) is a 20-item self-report measure designed to assess an individual's capacity for tolerating ambiguity. Each item on the RSAT is answered with "true" or "false," with higher scores indicating a greater tolerance for ambiguity. This scale evaluates an individual's ability to remain comfortable and function effectively in uncertain, complex, or ambiguous situations. | Mac Donald Jr, A. P. (1970). Revised scale for ambiguity tolerance: Reliability and validity. *Psychological Reports*, 26(3), 791-798. |
| Decision Satisfaction Scale (DSS)^a^ | The Decision Satisfaction Scale (DSS) is a 10-item self-report measure designed to assess an individual's level of satisfaction with a decision they have made. Each item on the DSS is rated on a 5-point Likert scale, ranging from 1 ("strongly disagree") to 5 ("strongly agree"). A higher total score on the DSS indicates a more positive attitude and greater satisfaction with the decision. | Sainfort F, Booske BC. Measuring post-decision satisfaction. *Medical Decision Making*. 2000;20:51061.  O’Connor AM. Validation of a decisional conflict scale. *Medical Decision Making*. 1995;15:25-30. |
| Health Literacy Screening Questions (HL)^a^ | The Health Literacy Screening Questions (HL) consist of three brief self-report items designed to assess patients' health literacy levels and the need for additional communication support. These questions evaluate how often patients experience difficulties understanding written medical information, requiring help reading healthcare materials, and confidence in filling out medical forms. Each question is rated on a 5-point Likert scale. | Chew LD, Bradley KA, Boyko EJ. Brief questions to identify patients with inadequate health literacy. *Family Medicine*. 2004;36(8):588-594. |
| Disclosure of Results to Others (DORTO)^a^ | A 5‐item self‐report measure determining to whom, if anyone, an individual disclosed their health results. | Ashida, S., et al. (2009). Disclosing the disclosure: Factors associated with communicating the results of genetic susceptibility testing for Alzheimer's disease. *Journal of Health Communication*, 14(8), 768-784. |
| University of North Carolina Genomic Knowledge Scale (GKS) | The Genomic Knowledge Scale (GKS), developed by Langer et al., is a self-report tool designed to evaluate individuals' comprehension of essential genomic concepts, aiding in informed decision-making regarding genomic sequencing. The scale features multiple-choice questions that address various topics, such as basic genetics, the purpose and implications of genomic testing, and ethical issues. Response options include "true," "false," and "not sure/I don't know" (included to minimise guessing). The GKS offers a quantitative assessment of genomic literacy, where higher scores reflect greater understanding. | Langer MM, Roche MI, Brewer NT, Berg JS, Khan CM, Leos C, et al. Development and Validation of a Genomic Knowledge Scale to Advance Informed Decision Making Research in Genomic Sequencing. *MDM Policy Pract*. 2017;2(1). |
| Strengths and Difficulties Questionnaire (SDQ) | The Strengths and Difficulties Questionnaire (SDQ) is a brief behavioural screening tool used to assess children and adolescents' emotional and behavioural attributes. It comprises 25 items divided into five subscales: Emotional Symptoms, Conduct Problems, Hyperactivity/Inattention, Peer Relationship Problems, and Prosocial Behavior. Each item is rated on a 3-point scale: "Not True," "Somewhat True," and "Certainly True." The questionnaire provides scores for each subscale as well as a total difficulties score, which is derived from the first four subscales and higher scores indicate more significant difficulties. The only exception is that lower scores on the Prosocial Behavior subscale suggest more significant difficulties and fewer prosocial behaviours. | Goodman R. The Strengths and Difficulties Questionnaire: A research note. *Journal of Child Psychology and Psychiatry*. 1997;38(5):581-586. |
| Vulnerable Child Scale (VCS)^a^ | The Vulnerable Child Scale (VCS) is a 16-item parent-report measure designed to assess parental perceptions of their child's vulnerability. Parents rate each item on a 4-point scale, with responses ranging from 1 ("definitely false") to 4 ("definitely true"). Two of the items are reverse-scored. The total scores range from 16 to 64, with lower scores indicating higher perceived vulnerability of the child. | Forsyth, B. W., et al. (1996). The child vulnerability scale: an instrument to measure parental perceptions of child vulnerability. *Journal of Pediatric Psychology*, 21(1), 89-10 1. |
| Genetic Essentialism^a^ | The Genetic Essentialism scale is a 6-item self-report questionnaire designed to measure participants' beliefs and perceptions about the influence of their genetic makeup. Each question is rated on a 4-point Likert scale, with responses ranging from 1 ("strongly disagree") to 4 ("strongly agree"). The scale aims to capture the extent to which individuals attribute characteristics and behaviours to genetic factors. Two questions from this scale were used in the PALM Study. | Klitzman, R., Appelbaum, P. S., Fyer, A., Martinez, J., Buquez, B., Wynn, J., & Chung, W. K. (2013). Researchers' views on return of incidental genomic research results: qualitative and quantitative findings. *Genetics in Medicine*, 15(11), 888-895.  Phelan, J. C. (2005). Geneticization of Deviant Behavior and Consequences for Stigma: The Case of Mental Illness. *Journal of Health and Social Behavior*, 46(4), 307-322. |
| Children with Special Health Care Needs Screener (CSHCNS) | The Children with Special Health Care Needs Screener (CSHCNS) is a five-item parent-report tool that identifies children who require special health care services due to chronic physical, developmental, behavioural, or emotional conditions. The screener asks parents about their child's use of prescription medications, the need for specialised therapies, increased need for medical or mental health services, functional limitations, and dependency on specialised healthcare services. Each item is scored based on the parent's responses, with options typically including "yes" or "no." | Bethell CD, Read D, Blumberg SJ, Newacheck PW, Stein RE, Strickland B, et al. *Identifying children with special health care needs: Development and evaluation of a short screening instrument*. Ambulatory Pediatrics. 2002;2(1):38-48. |
| ^a^ Denotes the same scales used in Desai et al. 2018. | | |

| **Supplementary Table 2. Perinatal outcomes** | | | | | | | | | | |
| --- | --- | --- | --- | --- | --- | --- | --- | --- | --- | --- |
|  | **Survey cohort (n=134)^a^** | | | | | **Clinical cohort (n=78)^a^** | | | | |
|  | **Controls (n=88)** | | **VUS (n=46)** | | **p** | **Controls (n=49)** | | **VUS (n=29)** | | **p** |
|  | **n** | **% (col)** | **n** | **% (col)** |  | **n** | **% (col)** | **n** | **% (col)** |  |
| **Infant sex** |  |  |  |  |  |  |  |  |  |  |
| Male | 36 | 40.9 | 26 | 56.5 | 0.09 | 22 | 44.9 | 15 | 51.7 | 0.56 |
| Female | 52 | 59.1 | 20 | 43.5 |  | 27 | 55.1 | 14 | 48.3 |  |
| **Baby head circumference percentile** |  |  |  |  |  |  |  |  |  |  |
| <3 | 1 | 1.4 | 0 | 0.0 | 0.25 | 1 | 2.2 | 0 | 0.0 | 0.32 |
| ≤ 3 or ≤97 | 62 | 87.3 | 33 | 78.6 |  | 38 | 84.4 | 20 | 74.1 |  |
| >97 | 8 | 11.3 | 9 | 21.4 |  | 6 | 13.3 | 7 | 25.9 |  |
| **Small for gestational age** |  |  |  |  |  |  |  |  |  |  |
| No | 77 | 88.5 | 44 | 95.7 | 0.17 | 45 | 91.8 | 27 | 93.1 | 0.84 |
| Yes | 10 | 11.5 | 2 | 4.4 |  | 4 | 8.2 | 2 | 6.9 |  |
| **Delivery** |  |  |  |  |  |  |  |  |  |  |
| Vaginal | 37 | 42.5 | 18 | 39.1 | 0.55 | 21 | 42.9 | 13 | 44.8 | 0.98 |
| Vaginal with forceps or vacuum | 13 | 14.9 | 10 | 21.7 |  | 8 | 16.3 | 5 | 17.2 |  |
| Emergency caesarean section | 11 | 12.6 | 8 | 17.4 |  | 6 | 12.2 | 4 | 13.8 |  |
| Elective caesarean section | 26 | 29.9 | 10 | 21.7 |  | 14 | 28.6 | 7 | 24.1 |  |
| **SCN or NICU admission** |  |  |  |  |  |  |  |  |  |  |
| Yes | 9 | 10.3 | 14 | 30.4 | 0.00 | 4 | 8.2 | 10 | 34.5 | 0.00 |
| No | 78 | 89.7 | 32 | 69.6 |  | 45 | 91.8 | 19 | 65.5 |  |
| **Milk type at discharge** |  |  |  |  |  |  |  |  |  |  |
| Breast milk | 60 | 69.0 | 27 | 58.7 | 0.42 | 35 | 71.4 | 17 | 58.6 | 0.50 |
| Formula | 4 | 4.6 | 4 | 8.7 |  | 2 | 4.1 | 2 | 6.9 |  |
| Breast milk and formula | 23 | 26.4 | 15 | 32.6 |  | 12 | 24.5 | 10 | 34.5 |  |
| **Hospital readmission within 28 days** |  |  |  |  |  |  |  |  |  |  |
| Yes | 4 | 4.6 | 6 | 13.0 | 0.04 | 3 | 6.1 | 5 | 17.2 | 0.04 |
| No | 82 | 94.3 | 37 | 80.4 |  | 46 | 93.9 | 22 | 75.9 |  |
| Not sure (review missing/not sure | 1 | 1.2 | 3 | 6.5 |  | 0 | 0.0 | 2 | 6.9 |  |
| **Medical reported medical issues at birth** |  |  |  |  |  |  |  |  |  |  |
| No | 76 | 86.4 | 28 | 60.9 | 0.001 | 41 | 83.7 | 17 | 58.6 | 0.01 |
| Yes | 12 | 13.6 | 18 | 39.1 |  | 8 | 16.3 | 12 | 41.4 |  |
| **Indication for prenatal diagnostic testing^b^** |  |  |  |  |  |  |  |  |  |  |
| Positive (‘high chance’) CFTS result | 42 | 48.3 | 10 | 21.7 | N/A | 23 | 46.9 | 7 | 24.1 | N/A |
| Positive (‘high chance’) NIPT result | 18 | 20.7 | 0 | 0.0 |  | 12 | 24.5 | 0 | 0.0 |  |
| Positive (‘high chance’) STSS result | 7 | 8.1 | 4 | 8.7 |  | 3 | 6.1 | 3 | 10.3 |  |
| Ultrasound abnormality | 0 | 0.0 | 26 | 56.5 |  | 0 | 0.0 | 16 | 55.2 |  |
| Other^c^ | 20 | 23.0 | 6 | 13.0 |  | 11 | 22.5 | 3 | 10.3 |  |
| ^a^ The survey cohort (n=134) included mothers who completed baseline questionnaires, while the clinical cohort comprised mother-child pairs who completed paediatric reviews and additional assessments. Comparisons found no differences between those who completed only online assessments and those who underwent further assessments. Missing values ranged from 1 to 15.  ^b^ CFTS; combined first trimester screening, STSS; second trimester serum screening, NIPT; non-invasive prenatal testing  ^c^ Other indication for prenatal diagnostic testing included: advanced maternal age (>36 years) (n=10), history of chromosomal condition (n=1), no clinical notes (n=1), suspected infection (n=6), maternal anxiety (n=3), paternal request (n=1), single gene testing (n=4 (negative cystic fib (n=2), negative DMD (n=1), unknown thalassaemia (n=1)).  See Supplementary Table 3 for details of ultrasound abnormalities | | | | | | | | | | |

| **Supplementary Table 3. VUS cases with an ultrasound abnormality reported prior to prenatal diagnostic testing (N=32)^a^** | | |
| --- | --- | --- |
| **ID** | **Gestation at prenatal diagnosis (weeks)** | **Ultrasound indication for prenatal diagnosis** |
| 1 | 21 | Absent nasal bone |
| 2 | 31 | Mild unilateral cerebral ventriculomegaly (12mm) |
| 3 | 23 | Hypoplastic nasal bone |
| 4 | 13 | Subcutaneous edema, nuchal translucency (3.2mm) |
| 5 | 21 | Agenesis of the corpus callosum |
| 6 | 34 | Right pleural effusion |
| 7 | 22 | Right sided aortic arch |
| 8 | 21 | Unilateral mild cerebral ventriculomegaly (11.5m) |
| 9 | 21 | Ventriculoseptal defect, suspected horseshoe kidney |
| 10 | 21 | Pulmonary stenosis |
| 11 | 24 | Ventriculoseptal defect, fetal growth restriction |
| 12 | 23 | Bilateral mild cerebral ventriculomegaly |
| 13 | 24 | Unilateral multicystic dysplastic kidney |
| 14 | 23 | Aberrant right subclavian artery |
| 15 | 25 | Atrioventricular septal defected |
| 16 | 21 | Unilateral cleft lip and palate, single umbilical artery, ambiguous genitalia |
| 17 | 23 | Unilateral cleft lip and palate |
| 18 | 20 | Bilateral talipes |
| 19 | 16 | Increased nuchal translucency (4.5mm), absent nasal bone |
| 20 | 16 | Increased nuchal translucency (4.2mm), dilated right jugular sac |
| 21 | 16 | Increased nuchal translucency (4.3mm) |
| 22 | 22 | Right sided aortic arch |
| 23 | 15 | Increased nuchal translucency |
| 24 | 16 | Increased nuchal translucency (3.1mm) |
| 25 | 15 | Increased nuchal translucency (4.2mm), dilated right jugular sac |
| 26 | 22 | Increased nuchal fold (8mm) |
| 27 | 22 | Cleft lip and palate |
| 28 | 13 | Increased nuchal translucency with dilated jugular sacs |
| 29 | 14 | Increased nuchal translucency (2.9mm) |
| 30 | 15 | Increased nuchal translucency (6.2mm) |
| 31 | 15 | Increased nuchal translucency (3.2mm) |
| 32 | 15 | Increased nuchal translucency (3.2mm) |

^a^ Note: *n=*32 comprised those with a structural anomaly (*n=*26), increased nuchal translucency measurement as part of a positive combined first trimester screening result (*n=*5) and cleft lip and palate with a positive second trimester serum screening result (*n=*1) (as shown in **Supplementary Table 2).**

| **Supplementary Table 4. Comparison of children with and without a VUS on measures of intellectual functioning, adaptive functioning, and behaviours associated with autism spectrum disorder, unadjusted and adjusted regression analysis results** | | | | | | | |
| --- | --- | --- | --- | --- | --- | --- | --- |
| **Measure** (Population Mean (SD)) | **Domain**  (Higher scores indicate higher levels of the domain measured) | **VUS (n=30)^a^**  Median (IQR)/Mean(SD) | **Control (n=50)^b^**  Median (IQR)/Mean(SD) | **Unadjusted** | | **Adjusted^e^** | |
|  |  |  |  | **β (95% CI)** | **p** | **β (95% CI)** | **p** |
| **WPPSI-IV /**  **WISC-V** (100 (15)) | Overall intellectual functioning | 96.3 (14.1) | 101.5 (16.0) | -0.005 (-0.012, 0.002) | 0.14 | -0.003 (-0.010, 0.005) | 0.52 |
|  | Working memory | 98.4 (16.6) | 101.2 (15.8) | -0.003 (-0.009, 0.004) | 0.47 | 0.000 (-0.008, 0.007) | 0.95 |
|  | Visual-spatial reasoning | 99.8 (14.6) | 101.2 (14.9) | 0.000 (-0.008, 0.007) | 0.95 | 0.001 (-0.007, 0.009) | 0.82 |
|  | Verbal comprehension | 95.5 (13.8) | 103.7 (14.6) | -0.009 (-0.016, -0.002) | 0.02 | -0.007 (-0.015, 0.001) | 0.08 |
|  | Fluid reasoning | 96.6 (17.7) | 97.9 (15.6) | -0.001 (-0.009, 0.006) | 0.75 | 0.001 (-0.007, 0.009) | 0.74 |
|  | Processing speed | 97.2 (13.5) | 99.8 (14.8) | -0.003 (-0.012, 0.006) | 0.49 | -0.003 (-0.012, 0.007) | 0.58 |
| **Vineland-3 Parent/Caregiver form** (100 (15)) | Overall adaptive functioning | 93.1 (13.9) | 92.2 (10.5) | 0.002 (-0.008, 0.011) | 0.73 | 0.002 (-0.008, 0.011) | 0.74 |
|  | Communication | 94.5 (82,102) | 94.5 (88,100) | 0.000 (-0.009, 0.010) | 0.93 | 0.002 (-0.008, 0.012) | 0.70 |
|  | Daily Living Skills | 93.7 (13.7) | 92.9 (13.3) | 0.001 (-0.007, 0.009) | 0.78 | 0.001 (-0.008, 0.009) | 0.91 |
|  | Socialization | 96.1 (13.9) | 95.9 (10.1) | 0.000 (-0.009, 0.010) | 0.94 | 0.001 (-0.009, 0.010) | 0.87 |
|  | Motor skills (gross and fine) | 96 (89, 102) | 96 (89,104) | -0.001 (-0.010, 0.007) | 0.74 | -0.002 (-0.011, 0.007) | 0.67 |
| **Vineland-3 Parent/**  **Caregiver form** Maladaptive scales^c^ | Internalizing behaviours (e.g., anxiety) | 17 (15,18) | 15 (14,18) | 0.011 (-0.028, 0.051) | 0.57 | 0.009 (-0.028, 0.046) | 0.63 |
|  | External behaviours (e.g., outbursts) | 17 (14,19) | 17 (14,18) | 0.014 (-0.027, 0.055) | 0.49 | 0.002 (-0.039, 0.044) | 0.91 |
| **SRS-2** (T-scores)^d^ | Overall score of behaviours associated with autism spectrum disorder | 51.5 (46,59.5) | 49 (43.5,55.5) | 0.008 (-0.002, 0.018) | 0.13 | 0.005 (-0.006, 0.017) | 0.34 |
|  | DSM-5 Compatible Scale: Social Communication and Interaction | 52 (44.5,61) | 48.5 (43.5,55.5) | 0.006 (-0.004, 0.017) | 0.23 | 0.005 (-0.006, 0.015) | 0.38 |
|  | DSM-5 Compatible Scale: Restricted Interests and Repetitive Behavior | 48 (45.5, 59.5) | 47 (43,55) | 0.004 (-0.006, 0.013) | 0.46 | 0.001 (-0.009, 0.011) | 0.86 |
| ^a^ The VUS sample size for each outcome ranged between n= 24 - 30.  ^b^ The control sample size for each outcome ranged between n= 39 - 50.  ^c^ Maladaptive behaviours (Vineland-3)* v-Scale score 0-24 range; Mean = 15; SD=3  ^d^ ≤59 = within normal limits (generally not associated with autism spectrum disorder); between 60-65 = mild range; between 66-75 = moderate range; ≥76 = severe range (strongly associated with clinical diagnosis of autism spectrum disorder)  ^e^ Maternal age at child's birth, maternal country of birth, maternal education level, maternal relationship status, income, and locality (metro/regional) | | | | | | | |

| **Supplementary Table 5. Clinical concerns on paediatric review** | | | | | |
| --- | --- | --- | --- | --- | --- |
|  | **Controls (n=49)** | | **VUS cases (n=29)** | | **p** |
|  | **n** | **%** | **n** | **%** |  |
| **Developmental domains** |  |  |  |  |  |
| **Growth** | 0 | 0% | 2 | 7% | 0.06 |
| **Development** | 13 | 27% | 10 | 35% | 0.46 |
| **Cognitive** | 4 | 8% | 6 | 21% | 0.11 |
| **Behavioural** | 16 | 33% | 10 | 34% | 0.87 |
| **Neurological** | 2 | 4% | 3 | 10% | 0.28 |
| **Medical domains** |  |  |  |  |  |
| **Blood** | 2 | 4% | 3 | 10% | 0.28 |
| **Cardiac** | 2 | 4% | 5 | 17% | 0.05 |
| **Dermatological** | 28 | 57% | 14 | 48% | 0.45 |
| **Dietary** | 9 | 18% | 6 | 21% | 0.80 |
| **Endocrine** | 0 | 0% | 2 | 7% | 0.06 |
| **Gastrointestinal** | 10 | 20% | 7 | 24% | 0.70 |
| **Hearing** | 2 | 4% | 7 | 24% | 0.01 |
| **Infection** | 6 | 12% | 11 | 38% | 0.01 |
| **Liver** | 0 | 0% | 1 | 3% | 0.19 |
| **Ocular** | 10 | 20% | 2 | 7% | 0.11 |
| **Orthopaedic** | 8 | 16% | 3 | 10% | 0.46 |
| **Pulmonary** | 8 | 16% | 9 | 31% | 0.13 |
| **Renal** | 6 | 12% | 5 | 17% | 0.54 |
| **Dysmorphic** |  |  |  |  | 0.12 |
| Yes | 2 | 4% | 5 | 17% |  |
| No | 44 | 92% | 22 | 76% |  |
| Possibly | 2 | 4% | 2 | 7% |  |

| **Supplementary Table 6. Comparison of mothers of children with and without a VUS on measures of maternal mental health and parenting and perceptions of their child’s behaviour and development** | | | | |
| --- | --- | --- | --- | --- |
| **Measure**  (Score range) | **Domain**  (Higher scores indicate higher levels of the domain measured) | **Mother of child with a VUS (N=46)^a^**  Median (IQR)/Mean(SD) | **Mother of child in Control group (N=88)^b^**  Median (IQR)/Mean(SD) | **p** |
| PHQ8 (0 – 24) | Depressive symptoms | 2.0 (0.0, 5.0) | 2.0 (1.0, 6.0) | 0.35 |
| STAI-S (0 – 80) | Short-term anxiety (state) | 27.5 (21.5, 37.5) | 34.0 (26.0, 44.0) | 0.08 |
| STAI-T (0 – 80) | Long-term anxiety (trait) | 34.0 (27.0, 42.0) | 36.0 (28.0, 47.0) | 0.20 |
| PSOC (0 – 102) | Parental competency levels | 76.0 (63.0, 87.0) | 71.0 (62.0, 81.0) | 0.38 |
| VCS (16 – 64) | Child vulnerability^d^ | 55.5 (50.0, 59.0) | 58.0 (50.0, 60.0) | 0.10 |
| DSS (10 – 50) | Satisfaction about having a prenatal microarray | 42.0 (39.0, 44.0) | 44.0 (40.0, 48.0) | 0.02 |
| SDQ (z-scores)^c^ | Total score | 0.1 (0.1, 0.8) | -0.4 (-0.9, 0.5) | 0.07 |
| SDQ subscales  (z-scores)^c^ | Emotional problems | 0.4 (-0.6, 0.8) | -0.3 (-0.9, 0.4) | 0.04 |
|  | Conduct problems | -0.2 (-0.9, 0.8) | -0.3 (-0.9, 0.3) | 0.26 |
|  | Hyperactivity | 0.0 (-0.8, 0.7) | -0.2 (-0.8, 0.3) | 0.23 |
|  | Peer problems | -0.2 (-0.8, 0.7) | -0.6 (-0.8, 0.4) | 0.21 |
|  | Prosocial behaviours | 0.0 (-0.5, 0.5) | 0.5 (-0.5, 1.0) | 0.15 |
| MFFS (0 - 48) | Family functioning | 20.0 (15.5, 24.0) | 18.0 (14.0, 23.0) | 0.36 |
| RSAT (0 - 20) | Uncertainty tolerance | 10.3 (3.4) | 10.6 (3.2) | 0.66 |
| GKS (0 - 25) | Genetic knowledge | 17.5 (13.0, 21.0) | 18.0 (14.0, 21.5) | 0.29 |
| ^a^ The VUS sample size for each outcome ranged between n= 40 - 46. ^b^ The Control sample size for each outcome ranged between n= 80 - 88. ^c^ A z-score is the number of standard deviations a given data point (X) is from the average and can be positive (+) or negative (-) in value. A z-score of 1 means the data point is 1 standard deviation above the average; a z-score of -1 means the point is 1 standard deviation below the average. ^d^ Higher scores indicate lower levels of child vulnerability. | | | | |

| **Supplementary Table 7. Measures of intellectual functioning, adaptive functioning, and behaviours associated with autism spectrum disorder between children with a no longer reported VUS and children without a VUS** | | | | |
| --- | --- | --- | --- | --- |
| **Measure** (Population Mean (SD)) | **Domain**  (Higher scores indicate higher levels of the domain measured) | **VUS (n=19)^a^**  Median (IQR)/Mean(SD) | **Control (n=50)^b^**  Median (IQR)/Mean(SD) | **p** |
| **WPPSI-IV / WISC-V** (100 (15)) | Overall intellectual functioning | 95.9 (14.7) | 101.5 (16.0) | 0.20 |
|  | Working memory | 99.1 (15.7) | 101.2 (15.8) | 0.64 |
|  | Visual-spatial reasoning | 102.3 (13.8) | 101.2 (14.9) | 0.79 |
|  | Verbal comprehension | 94.7 (15.9) | 103.7 (14.6) | 0.03 |
|  | Fluid reasoning | 94.8 (18.8) | 97.9 (15.6) | 0.52 |
|  | Processing speed | 100.6 (13.6) | 99.8 (14.8) | 0.85 |
| **Vineland-3 Parent/Caregiver form** (100 (15)) | Overall adaptive functioning | 93.9 (12.2) | 92.2 (10.5) | 0.56 |
|  | Communication | 96.0 (88.0, 102.0) | 94.5 (88.0, 100.0) | 0.68 |
|  | Daily Living Skills | 95.6 (11.4) | 92.9 (13.3) | 0.43 |
|  | Socialization | 96.3 (14.1) | 95.9 (10.1) | 0.91 |
|  | Motor skills (gross and fine) | 96.0 (~) | 96.0 (89.0, 104.0) | 0.97 |
| **Vineland-3 Parent/Caregiver form** Maladaptive scales^c^ | Internalizing behaviours (e.g., anxiety) | 16.0 (14.0, 18.0) | 15.0 (14.0, 18.0) | 0.97 |
|  | External behaviours (e.g., outbursts) | 18.0 (14.0, 19.0) | 17.0 (14.0, 18.0) | 0.53 |
| **SRS-2** (T-scores)^d^ | Overall score of behaviours associated with Autism Spectrum Disorder | 49.5 (46.0, 59.0) | 49.0 (43.5, 54.4) | 0.38 |
|  | DSM-5 Compatible Scale: Social Communication and Interaction | 48.0 (44.5, 61.0) | 48.5 (43.5, 55.5) | 0.74 |
|  | DSM-5 Compatible Scale: Restricted Interests and Repetitive Behavior | 48.0 (46.0, 57.5) | 47.0 (43.0, 55.0) | 0.26 |
| ^a^ The VUS sample size for each outcome ranged between n= 16 - 19. ^b^ The Control sample size for each outcome ranged between n= 39 - 50. ^c^ Maladaptive behaviours (Vineland-3)* v-Scale score 0-24 range; Mean = 15; SD=3 ^d^ ≤59 = within normal limits (generally not associated with autism spectrum disorder); between 60-65 = mild range; between 66-75 = moderate range; ≥76 = severe range (strongly associated with clinical diagnosis of autism spectrum disorder) | | | | |

| **Supplementary Table 8. Measures of intellectual functioning, adaptive functioning, and behaviours associated with autism spectrum disorder between children with a still reported/upgrade VUS and children without a VUS** | | | | |
| --- | --- | --- | --- | --- |
| **Measure** (Population Mean (SD)) | **Domain**  (Higher scores indicate higher levels of the domain measured) | **VUS (n=11)^a^**  Median (IQR)/Mean(SD) | **Control (n=50)^b^**  Median (IQR)/Mean(SD) | **p** |
| **WPPSI-IV / WISC-V** (100 (15)) | Overall intellectual functioning | 96.1 (14.0) | 101.5 (16.0) | 0.30 |
|  | Working memory | 96.6 (19.1) | 101.2 (15.8) | 0.41 |
|  | Visual-spatial reasoning | 94.5 (14.9) | 101.2 (14.9) | 0.18 |
|  | Verbal comprehension | 95.1 (9.3) | 103.7 (14.6) | 0.07 |
|  | Fluid reasoning | 99.9 (17.1) | 97.9 (15.6) | 0.76 |
|  | Processing speed | 91.9 (12.4) | 99.8 (14.8) | 0.17 |
| **Vineland-3 Parent/Caregiver form** (100 (15)) | Overall adaptive functioning | 91.8 (16.9) | 92.2 (10.5) | 0.92 |
|  | Communication | 88.0 (81.0, 102.0) | 94.5 (88.0, 100.0) | 0.67 |
|  | Daily Living Skills | 90.5 (17.1) | 92.9 (13.3) | 0.61 |
|  | Socialization | 95.9 (14.1) | 95.9 (10.1) | 0.99 |
|  | Motor skills (gross and fine) | 96.0 (92.0, 100.0) | 96.0 (89.0, 104.0) | 0.83 |
| **Vineland-3 Parent/Caregiver form** Maladaptive scales^c^ | Internalizing behaviours (e.g., anxiety) | 17.5 (15.0, 20.0) | 15.0 (14.0, 18.0) | 0.35 |
|  | External behaviours (e.g., outbursts) | 16.0 (14.0, 19.0) | 17.0 (14.0, 18.0) | 0.49 |
| **SRS-2** (T-scores)^d^ | Overall score of behaviours associated with Autism Spectrum Disorder | 52.0 (47.0, 63.0) | 49.0 (43.5, 54.4) | 0.23 |
|  | DSM-5 Compatible Scale: Social Communication and Interaction | 53.0 (48.0, 63.5) | 48.5 (43.5, 55.5) | 0.19 |
|  | DSM-5 Compatible Scale: Restricted Interests and Repetitive Behavior | 48.0 (43.5, 61.0) | 47.0 (43.0, 55.0) | 0.59 |
| ^a^ The VUS sample size for each outcome ranged between n= 8 - 11. ^b^ The Control sample size for each outcome ranged between n= 39 - 50. ^c^ Maladaptive behaviours (Vineland-3)* v-Scale score 0-24 range; Mean = 15; SD=3 ^d^ ≤59 = within normal limits (generally not associated with autism spectrum disorder); between 60-65 = mild range; between 66-75 = moderate range; ≥76 = severe range (strongly associated with clinical diagnosis of autism spectrum disorder) | | | | |
